# Pheophorbide a Derivatives Exert Antiwrinkle Effects on UVB-Induced Skin Aging in Human Fibroblasts

**DOI:** 10.3390/life11020147

**Published:** 2021-02-15

**Authors:** Hwa Lee, Ho-Yong Park, Tae-Sook Jeong

**Affiliations:** Industrial Bio-materials Research Center, Korea Research Institute of Bioscience and Biotechnology, Daejeon 34141, Korea; leehua@kribb.re.kr

**Keywords:** antiwrinkle, collagen, fibroblasts, pheophorbide a derivatives, matrix metalloproteinases

## Abstract

Pheophorbide a is a chlorophyll metabolic breakdown product. This study investigated the antiwrinkle effect of pheophorbide a (PA) and its derivatives, including pyropheophorbide a (PyroPA) and pyropheophorbide a methyl ester (PyroPA-ME), on ultraviolet (UV) B-stimulated CCD-986sk fibroblasts. PA, PyroPA, and PyroPA-ME effectively suppressed reactive oxygen species accumulation in UVB-exposed CCD-986sk fibroblasts. All three pheophorbides also reduced UVB-induced matrix metalloproteinase (MMP)-1 secretion and mRNA expression of *MMP-1*, *MMP-2*, and *MMP-9*. Treatment with pheophorbides resulted in increased procollagen synthesis, and this required enhancement of procollagen type I C-peptide content and mRNA expression of collagen type I alpha 1 (*COL1A1*) and *COL1A2* in CCD-986sk cells. These antiwrinkle effects were more potent with PA and PyroPA than with PyroPA-ME. Furthermore, PA and PyroPA suppressed UVB-induced phosphorylation of extracellular signal-regulated protein kinase and c-Jun N-terminal kinase but not p38. Moreover, all three pheophorbides inhibited NF-κB p65 phosphorylation. Therefore, these pheophorbides, especially PA and PyroPA, can be used as antiwrinkle agents, and PA- or PyroPA-rich natural resources can be used in functional cosmetics.

## 1. Introduction

Skin is the largest organ and accounts for approximately 10–15% of whole body weight in the human body [1]. The signs of aging are most visible on the skin, which comprises the epidermis, dermis, and subcutaneous tissue. As skin ages, the epidermis becomes thinner with a reduction in the proliferation of basal keratinocytes, and collagen production in the dermis decreases with the severity of photodamage [2]. Unlike most other organs, skin is directly exposed to ultraviolet (UV) rays from the sun. Photoaging, also known as extrinsic aging, mainly occurs in sunlight-exposed skin areas, such as the face. Photoaging is caused by chronic exposure to UV and accounts for more than 80% of facial aging [3]; it is characterized by clinically deep wrinkling, hyperpigmentation, laxity, and telangiectases [4]. UV radiation present in sunlight can be classified into three types based on wavelength, i.e., UVA (320−400 nm), UVB (280−320 nm), and UVC (200−280 nm). Among them, UVC is blocked by the ozone layer; however, it is the most mutagenic among the three UV types. UVB has more energy and is more mutagenic than UVA; it exerts its effects by directly damaging DNA [5]. In photoaged skin, UVB irradiation is predominantly related to wrinkle formation, while UVA irradiation is importantly related to skin sagging [6]. 

Oxidative stress induced by UV irradiation plays an important role in skin aging. The accumulation of reactive oxygen species (ROS)—originating as by-products of cellular oxidative metabolism or produced in response to UV irradiation—can induce the secretion and activation of matrix metalloproteinases (MMPs), which ultimately promote skin aging [7]. Activated MMPs can degrade collagen that is mainly synthesized by dermal fibroblasts in the extracellular matrix (ECM) of the dermis [8]. MMPs are calcium-dependent, zinc-containing metalloproteinases, and these are commonly classified into collagenases, gelatinases, stromelysins, matrilysins, membrane-type MMPs, and other MMPs [9]. MMP-1 is a collagenase that recognizes its substrate through a hemopexin-like domain and cleaves native fibrillar collagens [10]. MMP-2 and MMP-9 are gelatinases that degrade ECM components including types I and IV collagen. In this study, we used UVB-exposed CCD-986sk human fibroblasts to investigate activities of pheophorbides on regulation of wrinkle formation markers, including ROS, MMPs, and procollagen expression. 

Botulinum toxin and several synthetic products have been effectively used to prevent wrinkles, but these induce adverse events, such as allergic reactions [11], muscle weakness, and flu-like symptoms [12]. Naturally derived compounds have attracted much attention in the field of cosmetics due to their hypoallergenic and fast-absorbing properties. Many phytochemicals from natural products have been reported to repair wrinkles and prevent UV-induced skin aging [13]. Many herbal sources such as Aloe vera, *Curculigo orchioides* G., and green tea leaves as well as natural compounds inhibit collagenases and MMPs and exert antiwrinkle effects on human skin fibroblasts and human skin [14,15,16]. 

**Figure 1:**
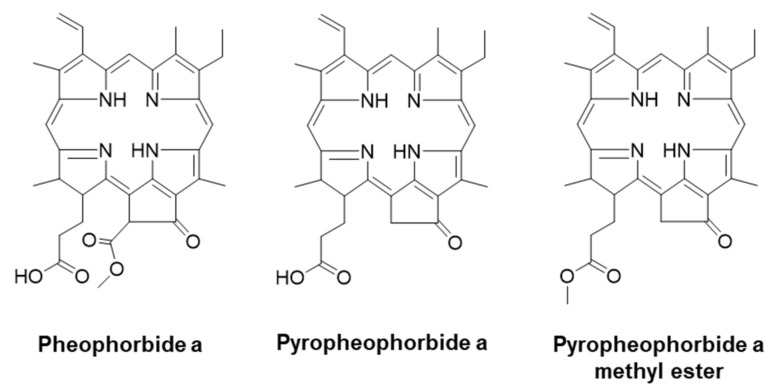
Chemical structures of three pheophorbides.

Pheophorbides are commonly produced by most photosynthetic plants and microalgae as breakdown products of chlorophyll upon senescence and/or fruit ripening [17]. Pheophorbide a (PA) exerts antiviral [18], antioxidant [19], anti-inflammatory [20], antidiabetic [21,22], anticancer [23], and antiparasitic effects [24] through regulation of hepatitis C virus, scavenging of the 2,2-diphenyl-1-picryldrazyl-radical, and induction of tumor necrosis factor alpha (TNFα) secretion, α-glucosidase activity, insulin secretion, cancer cell proliferation, and Leishmania amazonensis cell death, respectively. PA is a good photosensitizer and strongly absorbs light energy at 650−700 nm [25]. PA-mediated photodynamic therapy (PDT) exerts strong anticancer activity against various human cancer cells through ROS-mediated inhibition of phosphorylation of extracellular signal-regulated protein kinase (ERK) [25,26]. PA-mediated PDT showed strong efficacy, surpassing commercial Photofrin, on B16F10 skin melanoma [27]. In the absence of any photo-irradiation, PA is potentially cytotoxic to U87MC glioblastoma cells, but not toxic to normal HUVECs [28]. In addition, PA is reported to have antiwrinkle activity in UV-stimulated skin tissue through suppression of MMP-1 and MMP-3 expression (Granted patent: US 20170189292). Pyropheophorbide a (PyroPA), a decarboxylated derivative of PA, has been reported to inhibit the growth of hepatitis C virus [18] and influenza A [29]. PyroPA and pyropheophorbide a methyl ester (PyroPA-ME), based on its photosensitizing characteristics, can also be applied in anticancer PDT [30,31]. Further, PyroPA shows higher photobleaching efficiency than PyroPA-ME [32]. However, the antiwrinkle effects of PyroPA and PyroPA-ME have not been previously reported. Here, we surveyed and compared the antiwrinkle effects of PA and its derivatives, including PyroPA and PyroPA-ME (see structures in Figure 1) in UVB-exposed CCD-986sk human fibroblasts. Furthermore, the molecular mechanism underlying the effects of these pheophorbides was elucidated.

## 2. Results

### 2.1. Effects of Pheophorbides on the Viability of CCD-986sk Cells

**Figure 2:**
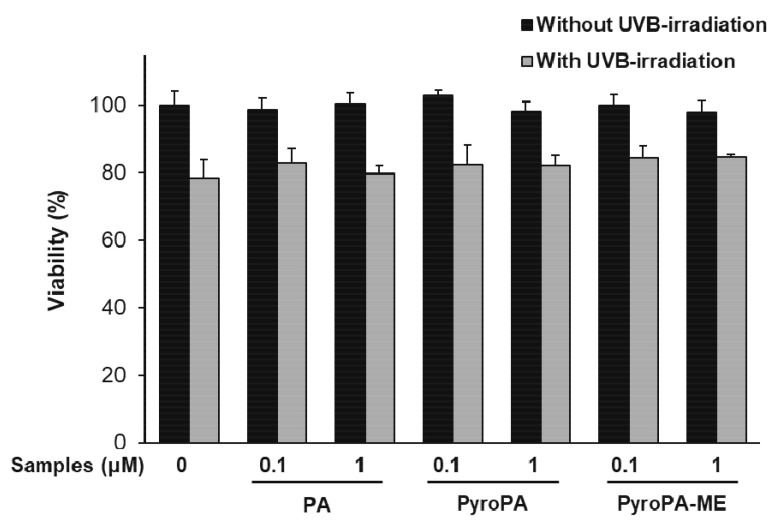
Cytotoxicity of pheophorbides towards CCD-986sk fibroblasts. Cells were treated with PA, PyroPA, and PyroPA-ME for 24 h with or without UVB irradiation. The mean ± S.D. (n = 3) is presented as data. PA, pheophorbide a; PyroPA, pyropheophorbide a; PyroPA-ME, pyropheophorbide a methyl ester.

Before determining the antiwrinkle effects of PA, PyroPA, and PyroPA-ME, we first examined their cytotoxicity. At concentrations up to 1 μM, none of the pheophorbides were toxic to CCD-986sk cells without or with UVB-irradiation (Figure 2). Subsequently, the antiwrinkle effects of the pheophorbides were assessed at concentrations of 0.1 and 1 μM.

### 2.2. Effects of Pheophorbides on ROS Accumulation in CCD-986sk Cells

**Figure 3:**
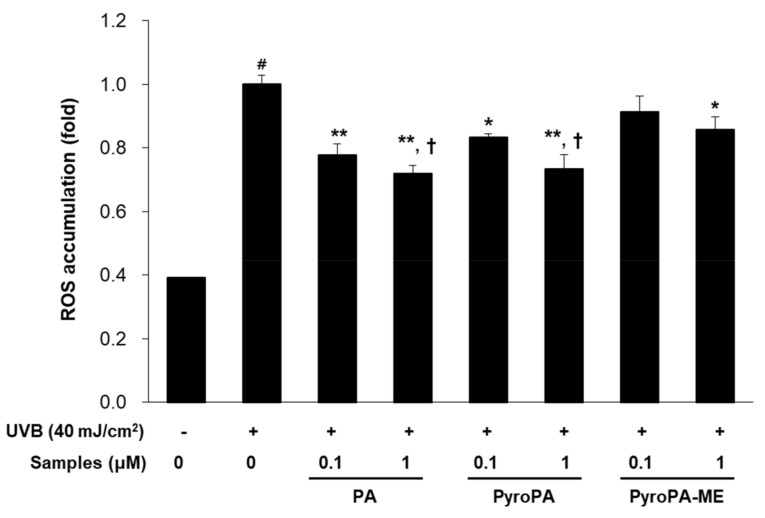
Effect of three pheophorbides on reactive oxygen species (ROS) accumulation in UVB-exposed CCD-986sk fibroblasts. Cells were treated with PA, PyroPA, and PyroPA-ME and exposed to UVB (40 mJ/cm^2^) for 24 h. ROS accumulation was determined based on 2′,7′-dichloro-fluorescin diacetate levels. The mean ± S.D. (n = 3) is presented as results. # *p* < 0.01 vs. cells not irradiated; * *p* < 0.05, ** *p* < 0.01 vs. cells irradiated with UVB only; and † *p* < 0.05 vs. cells treated with 1 μM PyroPA-ME.

To further investigate the antiwrinkle effects of PA, PyroPA, and PyroPA-ME in UVB-exposed CCD-986sk cells, levels of ROS were determined. UV exposure significantly increased ROS levels in CCD-986sk cells (Figure 3). However, treatment with 0.1 and 1 μM PA significantly reduced UVB-induced ROS accumulation by 36.5% and 46.2%, respectively, and treatment with 0.1 and 1 μM PyroPA significantly decreased ROS accumulation by 27.5% and 44.0%, respectively. In contrast, treatment with 0.1 μM PyroPA-ME did not significantly change UVB-induced ROS accumulation, whereas 1 μM PyroPA-ME reduced the ROS level by 23.5% (Figure 3). Briefly, among the three pheophorbides, PA and PyroPA showed stronger inhibitory activities with respect to ROS accumulation than that of the same concentration of PyroPA-ME in CCD-986sk cells.

### 2.3. Effects of Pheophorbides on MMP Expression in CCD-986sk Cells

**Figure 4:**
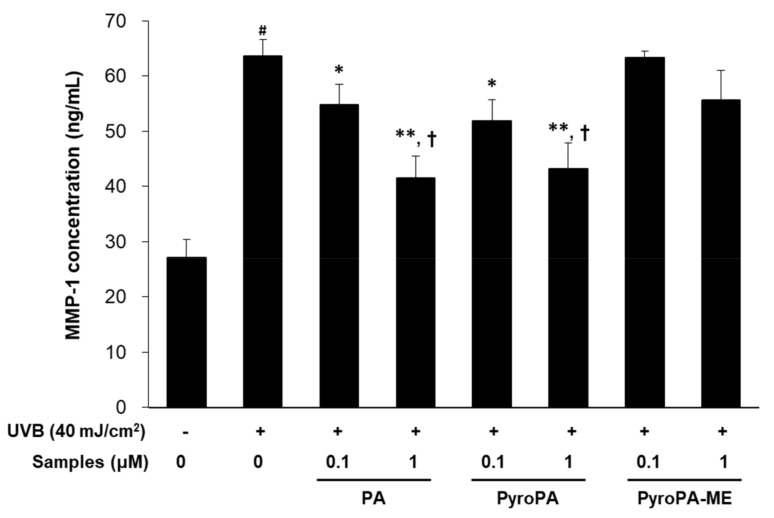
Effect of the three pheophorbides on matrix metalloproteinase (MMP)-1 levels in UVB-irradiated CCD-986sk fibroblasts. Cells were treated with PA, PyroPA, and PyroPA-ME and irradiated to UVB (40 mJ/cm^2^). After 24 h, MMP-1 protein concentrations were measured. The mean ± S.D. (n = 3) is presented as results. # *p* < 0.01 vs. cells not irradiated; * *p* < 0.05, ** *p* < 0.01 vs. cells irradiated with UVB only; and † *p* < 0.05 vs. cells treated with 1 μM of PyroPA-ME.

To determine whether pheophorbides exert inhibitory effects on MMP-1 expression in UVB-exposed CCD-986sk cells, protein levels of MMP-1 were measured. MMP-1 levels increased 2.3-fold after UVB exposure (Figure 4). Treatment with PA and PyroPA significantly inhibited UVB-induced MMP-1 upregulation, whereas that of equal concentrations of PyroPA-ME did not.

**Figure 5:**
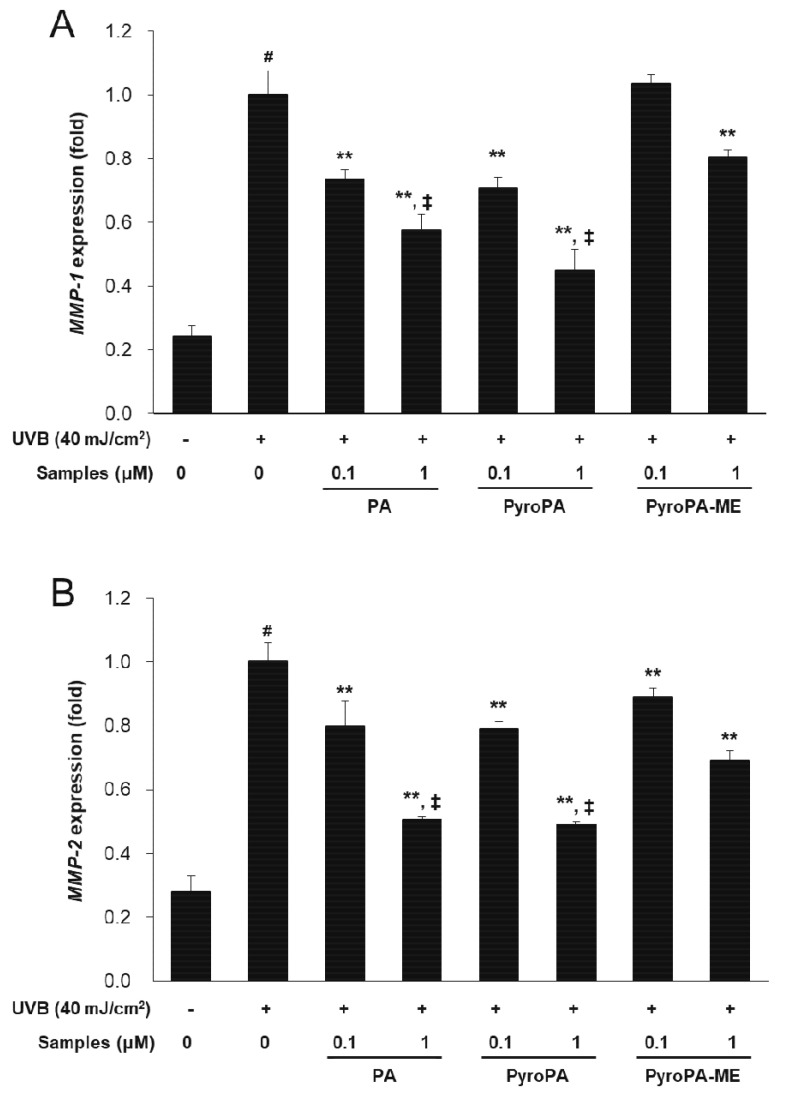

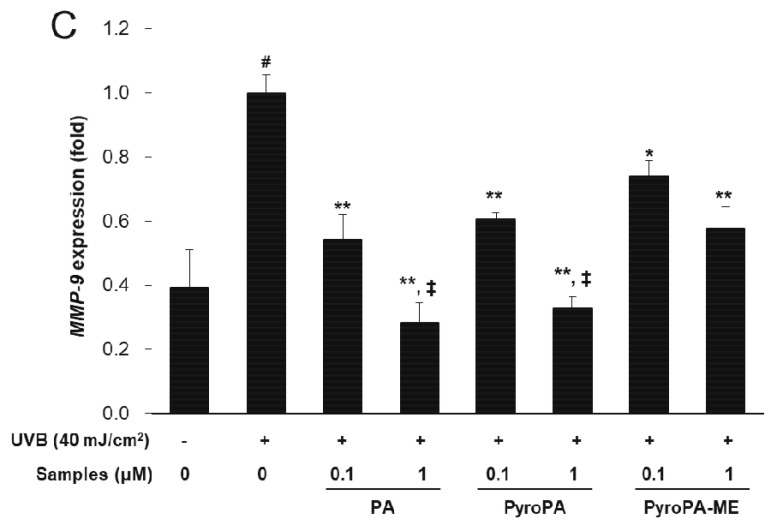
Effect of three pheophorbides on the expression of *MMP* mRNAs in UVB-exposed CCD-986sk cells. Cells were treated with PA, PyroPA, and PyroPA-ME and irradiated to UVB (40 mJ/cm^2^). The *MMP-1* (**A**), *MMP-2* (**B**), and *MMP-9* (**C**) expression levels were measured by real-time qRT-PCR and normalized against those of *ACTIN* (reference gene). The mean ± S.D. (n = 3) is presented as results. # *p* < 0.01 vs. cells not irradiated; * *p* < 0.05, ** *p* < 0.01 vs. cells irradiated with UVB only; and ‡ *p* < 0.01 vs. cells treated with 1 μM PyroPA-ME.

In addition, mRNA expression of *MMP-1*, *MMP-2*, and *MMP-9* was measured in UVB-irradiated CCD-986sk fibroblasts. Levels of all three mRNAs increased in UVB-irradiated CCD-986sk cells, but treatment with PA, PyroPA, or PyroPA-ME rescued this increase in all three transcript levels (Figure 5A–C). PA and PyroPA affected *MMPs* expression at a higher significance level than that of PyroPA-ME. These results imply that PA and PyroPA effectively inhibit wrinkle formation through inhibition of expression of *MMP* mRNAs expression in UVB-exposed CCD-986sk cells.

### 2.4. Effects of Pheophorbides on Collagen Expression in CCD-986sk Cells

**Figure 6:**
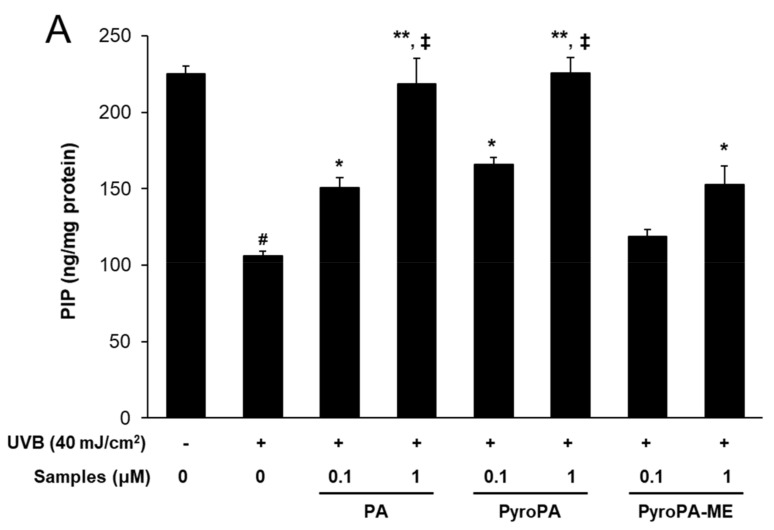

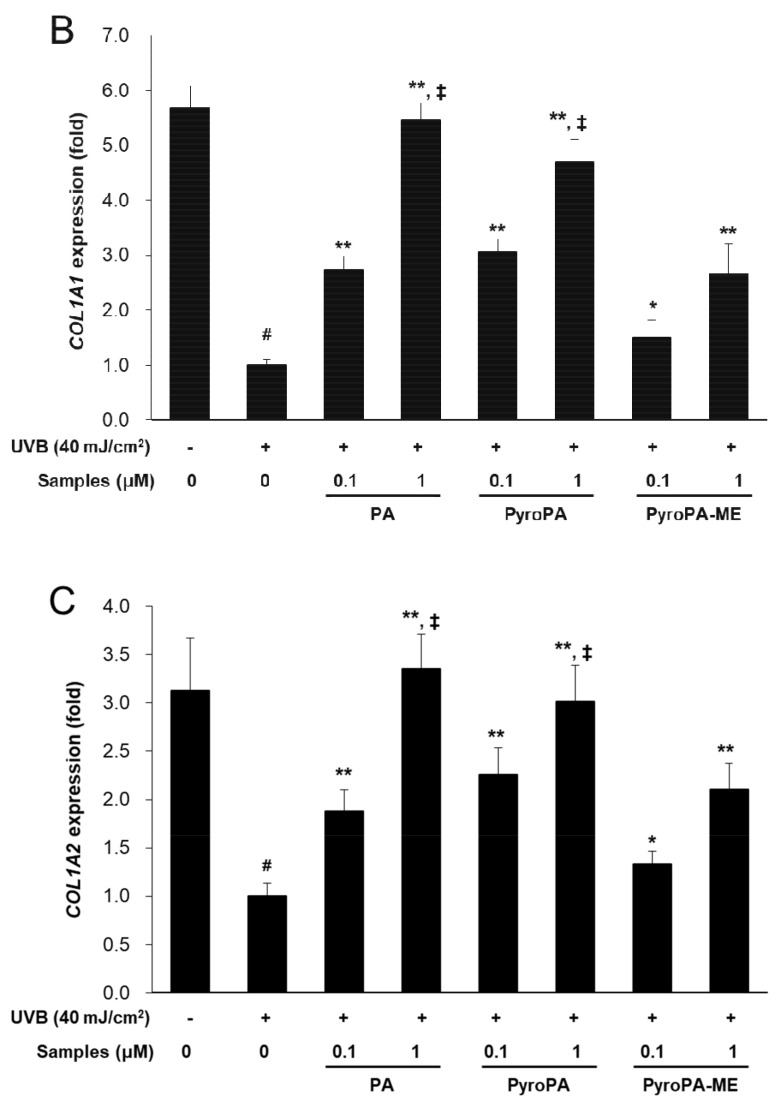
Effect of three pheophorbides on procollagen type I C-peptide (PIP) concentration and mRNA expression in UVB-induced CCD-986sk fibroblasts. Cells were treated with PA, PyroPA, and PyroPA-ME and irradiated to UVB for 40 mJ/cm^2^. (**A**) After 24 h, PIP concentrations were measured. (**B**,**C**) mRNA levels of *COL1A1* and *COL1A2* were measured by real-time qRT-PCR and normalized against that of *ACTIN* (reference gene). The mean ± S.D. (n = 3) is presented as results. # *p* < 0.01 vs. cells not irradiated; * *p* < 0.05, ** *p* < 0.01 vs. cells irradiated with UVB only; and ‡ *p* < 0.01 vs. cells treated with 1 μM PyroPA-ME.

Procollagen type I C-peptide (PIP) production in UVB-exposed CCD-986sk cells was measured using a PIP EIA kit. PIP concentration was significantly decreased after UVB exposure; however, treatment with PA, PyroPA, and PyroPA-ME significantly increased PIP levels compared to that of only UVB-treated cells (Figure 6A). Further, mRNA expression levels of procollagen synthesis-related genes collagen type I alpha 1 (*COL1A1*) and *COL1A2* increased upon treatment with PA, PyroPA, and PyroPA-ME (Figure 6B,C). These effects on collagen synthesis were more pronounced after treatment with PA and PyroPA than PyroPA-ME.

### 2.5. Effects of Pheophorbides on MAPK and NF-κB Signaling in CCD-986sk Cells

**Figure 7:**
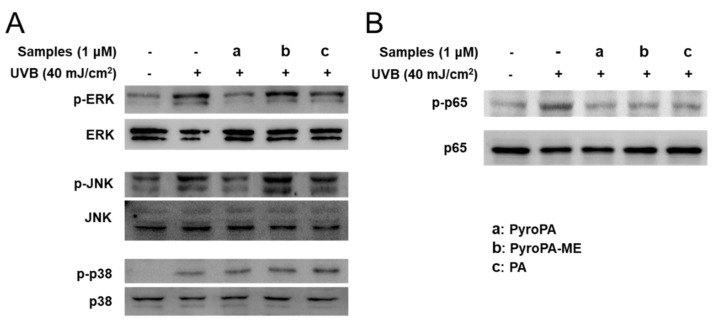
Effect of three pheophorbides on phosphorylation levels of mitogen-activated protein kinase (MAPK) and NF-κB signaling in UVB-induced CCD-986sk cells. Cells were treated with PA, PyroPA, and PyroPA-ME and exposed to UVB (40 mJ/cm^2^). Total and phosphorylation protein levels of ERK, JNK, and p38 MAPKs (**A**) and NF-κB p65 (**B**) were detected by immunoblotting.

In UVB-irradiation-induced skin aging, increased levels of intracellular ROS can enhance MMP expression by activating the MAPK and NF-κB pathways. Hence, to further investigate the mechanism of pheophorbide antiwrinkle effects, the phosphorylation levels of MAPK and NF-κB were measured after pheophorbide treatment in the background of UVB treatment. The phosphorylation levels of ERK and c-Jun N-terminal kinase (JNK) MAPKs, but not those of p38 MAPK, were markedly decreased by PA and PyroPA (Figure 7A). Furthermore, phosphorylation of NF-κB p65 markedly increased in response to UVB-irradiation; however, treatment with 1 μM PA, PyroPA, or PyroPA-ME markedly reversed the phosphorylation of NF-κB p65 (Figure 7B). These results suggest that PA and PyroPA can suppress UVB-induced MMPs expression and increase collagen synthesis through inhibition of ERK/JNK MAPKs and NF-κB signaling.

## 3. Discussion

Topical herbal treatment is an important therapy and exerts protective effects with respect to photoaging. Many plant and microalgae sources contain pheophorbide compounds. In the present study, we found that pheophorbides, including PA, PyroPA, and PyroPA-ME, exhibited antiwrinkle effects in UVB-irradiated CCD-986sk fibroblasts (Figure 3, Figure 4 and Figure 5). While all three compounds suppressed UVB-induced ROS accumulation and MMPs expression, PA and PyroPA showed more potent activities than PyroPA-ME at a treatment concentration of 1 μM. UVB-induced *MMP-1* expression levels were strongly decreased by treatment with PA, PyroPA, or PyroPA-ME up to 5 μM, with no difference among three pheophorbides (Figure S1).

UVB-irradiation directly damages intracellular DNA, resulting in the generation of thymine dimers and increased ROS production [33]. Increased ROS production can activate several signaling pathways related to cell proliferation, differentiation, senescence, inflammation, immunosuppression, and extracellular remodeling [34]. PA-mediated PDT causes cell apoptosis by generation of ROS in human oral squamous carcinoma cells and keloid fibroblasts with a decrease in type I collagen expression [26,35]. However, PA, PyroPA, and PyroPA treatment significantly decreased ROS production in UVB-exposed CCD-986sk cells (Figure 3). These divergent results might be due to different light irradiation (long wavelength vs. UVB) or cell conditions (abnormal cells vs. normal cells). Therefore, further studies are needed to elucidate the reasons for these divergent effects.

In humans, the dermis primarily contains ECM components. Collagen fibers present in the ECM play a role in strengthening skin and account for 75% of the dry weight of skin. In human skin, type I collagen accounts for 80–90% of the total collagen, whereas type III collagen accounts for 8–12% [36]. Procollagen produced by fibroblasts is secreted into the ECM and enzymatically altered to produce mature collagen in fibril form. UV-induced skin wrinkles occur as a result of an imbalance between ECM formation and MMPs production [37]. In UV-irradiated fibroblasts, the expression of *MMP-1*, *MMP-2*, *MMP-3*, and *MMP-9* time-dependently increase, resulting in photoaging [36]. Here, PA, PyroPA, and PyroPA-ME suppressed UVB-induced MMP-1 secretion and inhibited *MMP-1*, *MMP-2*, and *MMP-9* expression (Figure 4 and Figure 5). Moreover, the three pheophorbides increased collagen synthesis by increasing PIP content and *COL1A1* and *COL1A2* expression (Figure 6). Therefore, all three pheophorbides exert antiwrinkle effects by inhibiting *MMP-1*, *MMP-2*, and *MMP-9* expression and concurrently enhancing collagen synthesis in skin fibroblasts.

MAPK signaling plays an important role in mediating photoaging in UVB-exposed fibroblasts [38]. UVB-irradiation-induced increases in oxidative stress enhance the phosphorylation of ERK, JNK, and p38 to activate MAPK signaling. These changes activate activator protein 1 (AP-1) by phosphorylation of c-Jun and c-Fos and further increase collagen degradation by enhancing MMPs expression in UVB-irradiated fibroblasts [39]. However, activated AP-1 inhibits transforming growth factor β signaling, suppressing the procollagen promoter via phosphorylation of Smad [40]. Bui-Xuan et al. reported that photo-activated PA enhances the apoptosis of human breast adenocarcinoma cells by suppressing ERK phosphorylation [41]. In the present study, PA suppressed ERK MAPK (Figure 7), confirming the previous report. In addition to ERK, PA or PyroPA treatment inhibited JNK phosphorylation, thereby inhibiting MAPK signaling induced by UVB-irradiation. NF-κB, a transcription factor, can also be activated by UVB-induced oxidative stress [42]. NF-κB signaling is initiated upon activation of I-κB kinase and subsequent translocation of NF-κB to the nucleus, where it modulates expression of inflammatory cytokines, comprising interleukin (IL)-1β, IL-8, IL-6, and TNFα in response to UV irradiation [43]. Activated NF-κB is responsible for upregulation of MMPs in dermal fibroblasts [44]. Suppressing NF-κB signaling by using an inhibitor can protect against UVB-induced photoaging [45]. Heinrich et al. reported that PA treatment markedly suppressed the activation of NF-κB in PMA-induced HeLa cells [46]. In the current study, all three pheophorbides markedly suppressed the phosphorylation of NF-κB p65. These results confirmed that PA treatment strongly inhibits activation of NF-κB, and further indicate that another pheophorbide derivative, PyroPA, strongly inhibits UVB-induced activation of MAPKs and NF-κB pathways to protect against photoaging. Here, PA and PyroPA exerted a more potent effect on ROS accumulation, MMP-1 secretion, and procollagen synthesis than that of PyroPA-ME in UVB-irradiated fibroblasts. PyroPA-ME is a methylated form of PyroPA; the presence of the hydroxyl group in PA and PyroPA, which is absent in PyroPA-ME, is very important to their antiwrinkle activity (Figure 1). In fact, these pheophorbides may be used in combination to treat wrinkles, while using herbal sources. Therefore, further studies are needed to investigate the effects of combination treatments of PA and its derivatives on skin photoaging.

In summary, three pheophorbides, PA, PyroPA, and PyroPA-ME, present in many photosynthetic plants and microalgae, showed antiwrinkle activities in UVB-exposed CCD-986sk cells. All three pheophorbides decreased UVB-induced ROS production and *MMP-1*, *MMP-2*, and *MMP-9* expression. In contrast, all three pheophorbides increased procollagen levels and expression of *COL1A1* and *COL1A2* in UVB-exposed CCD-986sk cells. This is the first report of the molecular mechanism underlying antiwrinkle effects of PyroPA and PyroPA-ME. Moreover, PA and PyroPA were found to be more effective antiwrinkle agents than PyroPA-ME. Furthermore, we showed that the antiwrinkle activities were achieved by suppression of MAPK and NF-κB signaling pathways. Therefore, the use of photosynthetic plant leaves or microalgae as sources of antiwrinkle compounds might produce beneficial effects owing to the presence of pheophorbides, such as PA, PyroPA, and PyroPA-ME. According to the results of this study, pheophorbide compounds, especially PA and PyroPA, can be used as potential therapeutic ingredients to treat skin photoaging.

## 4. Materials and Methods

### 4.1. Chemicals

PyroPA was obtained from Cayman Chemical (Ann Arbor, MI, USA). PA and PyroPA-ME were purchased from Toronto Research Chemicals (Toronto, ON, Canada).

### 4.2. Cell Culture and Cell Viability Assay

The CCD-986sk human skin fibroblasts were purchased from the Korean Cell Line Bank (Seoul, Korea) and cultured in Iscove’s Modified Dulbecco’s Medium (IMDM; Welgene Inc., Gyeonsan, Korea) containing 10% fetal bovine serum (FBS; Hyclone, Logan, UT, USA), 1% Penicillin-Streptomycin Solution (Hyclone) at 37 °C in a humidified incubator with air containing 5% CO_2_.

For measurement of cell viability, CCD-986sk fibroblasts were plated in 96-well plates and incubated overnight and then treated with pheophorbide compounds for 24 h. UVB irradiation was conducted using a UVB lamp (Sankyo-Danki, Kanagawa, Japan) that emits wavelengths from 280 nm to 360 nm and peaked at 305‒310 nm. Cell viability was detected using the D-Plus™ CCK kit (Donginls, Daejeon, Korea).

### 4.3. Measurement of ROS Accumulation

CCD-986sk fibroblasts were plated in 96-well plates. After adhesion to plates, fibroblasts were treated with the indicated concentration of PA, PyroPA, and PyroPA-ME. After 2 h, fibroblasts were incubated with 20 μM 2′,7′-dichloro-fluorescin diacetate (Thermo Fisher Scientific, Waltham, MA, USA) at 37 °C for 60 min. The cells were irradiated with 40 mJ/cm^2^ UVB and then incubated for 60 min. Fluorescence intensities were measured using a Wallac-1420 spectrofluorometer (Perkin-Elmer, Turku, Finland) at 485 nm excitation and 535 nm emission wavelengths.

### 4.4. Detection of MMP-1 and Procollagen Concentration

CCD-986sk fibroblasts were stimulated with 40 mJ/cm^2^ UVB and then treated with the pheophorbide compounds (0.1 and 1 μM) for 24 h. MMP-1 and PIP were measured using the MMP-1 ELISA kit (Abcam, Cambridge, MA, USA) and PIP EIA kit (Takara Bio Inc., Shiga, Japan), respectively.

### 4.5. Real-Time Quantitative RT-PCR (qRT-PCR)

CCD-986sk fibroblasts were plated in 6-well plates, exposed to UVB (40 mJ/cm^2^) and then treated with 0.1 and 1 μM of PA, PyroPA, and PyroPA-ME for 24 h. Total RNA was prepared using TRI (Ambion, Carlsbad, CA, USA) and synthesized cDNA using a cDNA synthesis kit (Applied Biosystems, Foster City, CA, USA). A 7500 Real-Time PCR system (Life Technologies, Grand Island, NY, USA) was used to conduction of qRT-PCR with SYBR Mastermix (Roche, Mannheim, Germany). The used primers are shown in Table 1.

### 4.6. Immunoblotting

Anti-p-ERK, anti-ERK, anti-p-JNK, anti-p-p38, anti-p-NF-κB p65, and anti-NF-κB p65 antibodies (Cell Signaling Technology, Danvers, MA, USA) and anti-JNK and anti-p38 antibodies (Santa Cruz Biotechnology, Santa Cruz, CA, USA) were used to detect specific proteins. Protein expression was determined using a Chemiluminescent HRP Substrate (Merck Millipore, Burlington, MA, USA) and a LAS-4000 luminescent-image reader (Fuji Photo Film, Tokyo, Japan). Image MultiGauge (Fuji Photo Film) was used to analyze immunoreactive signals.

### 4.7. Statistical Analysis

Data were expressed as the mean ± standard deviations (S.D.) and analyzed using Student’s *t*-test to assess the differences between treatment-groups. A *p*-value < 0.05 was considered significant.

## Figures and Tables

**Table 1:** Primers sequences used for qRT-PCR.

Gene (NCBI Reference Sequence)	Forward Primer	Reverse Primer
*ACTIN (NM_001101)*	GGCACCACACCTTCTACAAT	GCCTGGATAGCAACGTACAT
*COL1A1 (NM_000088)*	TGGCCTCGGAGGAAACTTT	GCTTCCCCATCATCTCCATTC
*COL1A2 (NM_000089)*	CGGTGGTGGTTATGACTTTGGT	GAAGGGTCTCAATCTGGTTGTTG
*MMP-1 (NM_001145938)*	AACACATCTGACCTACAGGATTGAAA	CTTGGTGAATGTCAGAGGTGTGA
*MMP-2 (NM_001127891)*	ACTGGAGCAAAAACAAGAAGACATAC	TCCATTTTCTTCTTCACCTCATTG
*MMP-9 (NM_004994)*	GCGCTGGGCTTAGATCATTC	GTGCCGGATGCCATTCA

## Data Availability

The data presented in this study are available on request from the corresponding authors.

## Supplementary Materials

The following are available online at https://www.mdpi.com/2075-1729/11/2/147/s1, Figure S1: Effects of the three pheophorbides on *MMP-1* mRNA expression in UVB-exposed CCD-986sk fibroblasts.
