# Supplementary material for: Pheophorbide a Derivatives Exert Antiwrinkle Effects on UVB-Induced Skin Aging in Human Fibroblasts
Source: Life (Basel). 2021 Feb 15;11(2):147. doi: 10.3390/life11020147 (PMC7918994; doi:10.3390/life11020147)
Supplement: Supplementary file 1 [file life-11-00147-s001.pdf]

Supplementary File

Pheophorbide a Derivatives Exert Anti-wrinkle Effects on UVB-induced Skin Aging in Human Fibroblasts

Hwa Lee †, Ho-Yong Park *, and Tae-Sook Jeong *

Industrial Bio-materials Research Center, Korea Research Institute of Bioscience and Biotechnology, Daejeon 34141, Republic of Korea; leehua@kribb.re.kr (H.L.); hypark@kribb.re.kr (H.-Y.P); tsjeong@kribb.re.kr (T.-S.J)

† Earlier known as Hua Li

* Correspondence: tsjeong@kribb.re.kr (T.-S.J); hypark@kribb.re.kr (H.-Y.P); Tel.: +82-42-860-4558 (T.-S.J.); +82-42-860-4650 (H.-Y.P.)

Treatments with 5 μM of PA, PyroPA, and PyroPA-ME were strongly decreased UVB-induced *MMP-1* expression levels, with no difference among three pheophorbides.

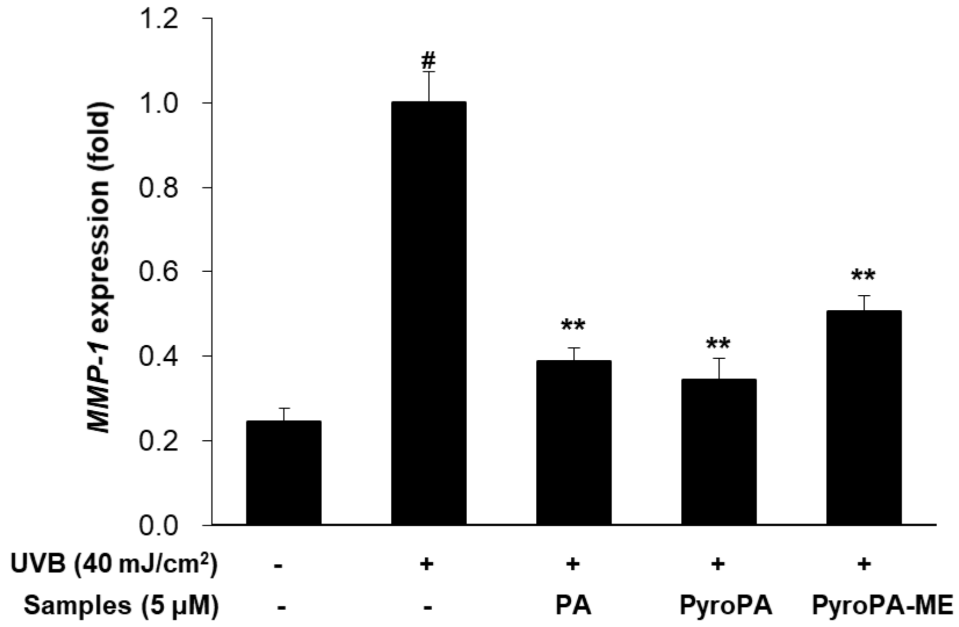

Figure S1. Effects of the three pheophorbides on the expression of *MMP-1* mRNA in UVB-exposed CCD-986sk cells. Cells were treated with 5 μM pheophorbides and exposed to UVB (40 mJ/cm^2). The mRNA levels of *MMP-1* were measured by real-time qRT-PCR and normalized using *ACTIN* as a reference gene. Values are presented as the mean ± SD (n = 3). #$p < 0.01$ vs. cells treated with media only; **$p < 0.01$ vs. cells treated with UVB only.
